# Aging-induced multi-scale study on the interfacial adhesion mechanism of steel slag and RAP recycled asphalt mixtures

**DOI:** 10.1371/journal.pone.0350936

**Published:** 2026-07-15

**Authors:** Yaodong Zhang, Yanan Cui, Shun Guo, Zhiyong Li

**Affiliations:** 1 School of Mechanics and Aeronautics of Inner Mongolia University of Technology, Hohhot, China; 2 Inner Mongolia Key Laboratory of Green Construction and Intelligent Operation and Maintenance of Civil Engineering, Inner Mongolia University of Technology, Hohhot, China; 3 Institute of Civil Engineering, Inner Mongolia University of Technology, Hohhot, China; China Construction Fourth Engineering Division Corp. Ltd, CHINA

## Abstract

To investigate the interfacial adhesion mechanism of steel slag and reclaimed asphalt pavement (RAP) dual solid waste recycled asphalt mixtures under aging conditions, the chemical and mineral properties of three aggregates (basalt, steel slag, and RAP) were characterized using XRF, XRD, and SEM-EDS. Interfacial adhesion behavior between asphalt and aggregates was studied through contact angle tests, AFM, and pull-off tests, and the evolution of interfacial properties during secondary aging of reclaimed asphalt was analyzed. The results show that Cl and S elements detected in the residual aged asphalt film on RAP aggregates represent the chemical imprint of the service environment. Chemical bonding occurs between steel slag and asphalt, forming a reaction transition layer, while basalt is dominated by physical adsorption with a clear and dense interface. Although the adhesion work of steel slag (69.12 mJ/m²) is slightly lower than that of basalt (71.01 mJ/m²), its pull-off strength (2.48 MPa) is higher due to mechanical interlocking from its rough and porous surface, revealing a synergistic enhancement mechanism. During reclaimed asphalt re-aging, functional group indices and micromorphology exhibit regular evolution, with 30h as the critical threshold for performance degradation. Adhesion work, AFM adhesion force, and pull-off strength show consistent positive correlations (R² = 0.92 and 0.89), forming a multi-scale interfacial performance evaluation system from thermodynamics to macro-mechanics.

## 1. Introduction

With the in-depth advancement of the “dual carbon” strategy, the resource utilization of industrial solid waste and road waste materials has become a research hotspot in the field of road engineering. As a byproduct of steelmaking, China’s annual steel slag emission exceeds 100 million tons, with a comprehensive utilization rate of less than 30% [1]. The recycling of reclaimed asphalt pavement (RAP) aggregate materials also faces performance bottlenecks at high mixing ratios [2]. The synergistic application of steel slag and RAP in asphalt mixtures can both achieve solid waste resource utilization and leverage their complementary advantages. Steel slag’s highly alkaline minerals provide chemical bonding sites for asphalt [3], while the aged asphalt carried by RAP can restore its bonding performance through rejuvenation [4]. However, the interfacial adhesion mechanism of the dual solid waste system remains unclear, particularly the synergistic effect between the chemical activity of steel slag and the residual aged asphalt film of RAP aggregates, which urgently requires systematic investigation.

The interfacial adhesion between asphalt and aggregates is key to determining the pavement performance of mixtures [5]. Surface energy theory is an important method for evaluating interfacial adhesion performance. Little and Bhasin systematically elaborated the quantitative relationship between surface energy parameters and adhesion work, and Tan and Guo [6] further validated the applicability of surface energy theory in asphalt-aggregate systems. XRD and XRF techniques can reveal the mineral phases and chemical compositions of aggregates. Horgnies et al. [7] used these methods to confirm the decisive role of aggregate surface chemical composition on interfacial adhesion. SEM-EDS can directly observe interfacial morphology and element distribution, and Zhang et al. [8] discovered the microscopic penetration behavior of asphalt on aggregate surfaces using this method. AFM can analyze asphalt microstructure from the nanoscale, and Nahar et al. [9] observed the “three-layer structure” at the interface between RAP and virgin asphalt.

Basalt, as a typical basic rock material, is widely used as a reference aggregate for asphalt mixtures due to its balanced chemical composition and abundant surface active sites [10]. Zhou et al. found through surface energy theory that the adhesion work between basalt and asphalt mainly originates from the dispersive component contribution, with its interfacial adhesion dominated by physical adsorption and polar interactions. Zhang et al. [8] observed using SEM that asphalt can penetrate into micro-pits and micro-cracks on the basalt surface, forming a mechanical interlocking structure, with no obvious chemical reaction product layer at the interface. Habal and Singh [11] confirmed through pull-off tests that the tensile strength of the basalt-asphalt interface is positively correlated with its surface energy parameters. Unlike the chemical bonding-dominated steel slag and the residual film activation-dominated RAP aggregates, the interfacial adhesion of basalt can be classified as physical adsorption and mechanical interlocking-dominated.

The interfacial adhesion mechanism between steel slag and asphalt has been a research hotspot in recent years. Chen et al. [12] found that alkaline minerals (CaO, Fe₂O₃) in steel slag can undergo saponification reactions with acidic components in asphalt, generating carboxylate interfacial compounds. Hu et al. [13] further confirmed this chemical bonding mechanism and found through XRD analysis that the RO phase content on the steel slag surface significantly decreased after asphalt coating. Suryawanshi et al. [14] pointed out that the rough and porous surface morphology of steel slag provides additional mechanical interlocking contribution, making its interfacial strength exceed that of pure chemical adsorption. Zhang et al. [8] revealed the phenomenon of intensified lattice distortion after steel slag coating from the perspective of micro-strain, providing quantitative evidence for chemical bonding.

The interfacial behavior of RAP aggregates is distinctive due to the residual aged asphalt film on its surface. Nahar et al. [9] observed three distinct layers at the RAP aggregate-asphalt interface: mineral substrate, intermediate residual layer, and new asphalt layer, confirming the existence of the residual film and its influence on interfacial behavior. Yang et al. [15] studied the diffusion behavior of rejuvenators in the aged film, finding that rejuvenators can effectively restore the bonding performance of aged asphalt. Ren et al. [16] revealed from a molecular dynamics perspective the microscopic mechanism of rejuvenator molecules penetrating the aged asphalt network and causing its swelling, explaining the phenomenon of increased interplanar spacing after RAP aggregate coating.

Recycled asphalt mixtures undergo re-aging during actual service, and their chemical structure and interfacial adhesion performance evolve over time. Existing studies have shown that aging leads to increased polar functional groups in asphalt and changes in molecular weight distribution, thereby affecting the interfacial bonding ability between asphalt and aggregates [8,16]. However, the evolution pattern of interfacial adhesion behavior during the re-aging process of reclaimed asphalt remains unclear, particularly the evolution mechanism of the interaction between rejuvenators and aged asphalt after long-term aging, which lacks systematic research.

In summary, existing studies have separately explored the adhesion mechanisms of basalt, steel slag, and RAP aggregates with asphalt interfaces. However, most existing studies focus on single aggregates, lacking systematic comparison of the interfacial adhesion of three aggregates: basalt, steel slag, and RAP aggregates. Moreover, insufficient attention has been paid to the evolution pattern of interfacial performance during the re-aging process of reclaimed asphalt. To truly reflect the particle size distribution, mixing ratio of aggregates, and preparation process of reclaimed asphalt in engineering practice, this study determines the optimal mixing ratio through mix design, and on this basis, prepares the aggregates and asphalt samples required for interfacial adhesion tests. Through XRF, XRD, SEM-EDS, contact angle tests, AFM, and pull-off tests, it systematically reveals the interfacial adhesion modes of three aggregates and the evolution pattern of interfacial performance during the re-aging process of reclaimed asphalt, establishing a multi-scale interfacial performance evaluation system from thermodynamics to macro-mechanics.

## 2. Raw materials and test methods

### 2.1 Raw materials

#### 2.1.1 RAP.

RAP aggregates was taken from the reconstruction project of Provincial Highway 311 in Wuchuan County, Hohhot, Inner Mongolia. The original pavement aggregate was granite. The average old asphalt content of RAP coarse aggregates was determined to be 3.78% using the centrifugal extraction method. The basic properties of the aged asphalt after extraction are shown in Table 1.

**Table 1:** Properties of aged asphalt after RAP extraction are presented.

Test Index	Test Result	Technical Requirement for 90# Base Asphalt
Penetration (25°C, 0.1 mm)	17.7	80 ⁓ 100
Softening point (°C)	52.1	>42
Ductility at 10°C (cm)	6.19	>20
Brookfield viscosity at 135°C (Pa·s)	1.139	<3

*Footnotes: The RAP-extracted asphalt is aged asphalt recovered from field RAP. Its lower performance compared to 90# base asphalt is expected due to long-term field aging.*

#### 2.1.2 Steel slag.

Steel slag was taken from the converter steel slag of Baotou Steel Group in Inner Mongolia, aged for about 2 years. The steel slag has rich edges and corners and a porous surface, typical characteristics of metallurgical slag.

#### 2.1.3 Basalt.

Basalt was taken from a quarry around Hohhot and used as a reference aggregate.

#### 2.1.4 Asphalt and rejuvenator.

90# base asphalt was selected as the new asphalt, and LY-ZS aromatic extract was selected as the rejuvenator. Their basic properties are shown in Tables 2 and 3.

**Table 2:** Properties of new asphalt (90# base asphalt).

Test Index	Test Result	Technical Requirement for 90# Base Asphalt
Penetration (25°C, 0.1 mm)	86	80 ⁓ 100
Softening point (°C)	45.3	>42
Ductility at 10°C (cm)	75.3	>20

**Table 3:** Basic performance indexes of rejuvenator.

Test Item	Test Result	Performance Index
Viscosity at 60°C (Pa·s)	168	50-175
Aromatic content (%)	76.5	>60
Saturated content (%)	22	≤30
Flash point (°C)	275	>220
Density at 15°C (g/cm³)	0.98	0.95-1.1

### 2.2 Mix design

The interfacial adhesion studied in this paper involves the interaction between three aggregates (basalt, steel slag, RAP aggregates) and asphalt. The aggregates, asphalt, and rejuvenator used all originate from the actual mix proportion of AC-16 recycled asphalt mixture. To truly reflect the particle size distribution, mixing ratio of aggregates, and preparation of reclaimed asphalt in engineering practice, this section first introduces the mix design of the mixture to clarify the source and preparation method of materials for subsequent interfacial adhesion tests.

#### 2.2.1 Reference aggregate gradation.

The synthetic gradation of AC-16 reference aggregate is shown in Table 4.

**Table 4:** Synthetic gradation of AC-16 aggregate.

Sieve size (mm)	19	16	13.2	9.5	4.75	2.36	1.18	0.6	0.3	0.15	0.075
Synthetic gradation (%)	100	94.5	81.2	66.4	45.9	30.2	20.8	13.7	9.0	6.5	4.7
Upper limit (%)	100	100	92	80	62	48	36	26	18	14	8
Lower limit (%)	100	90	76	60	34	20	13	9	7	5	4

*Footnotes: The synthetic gradation meets the AC-16 gradation requirements specified in JTG F40-2004.*

**Fig 1:**
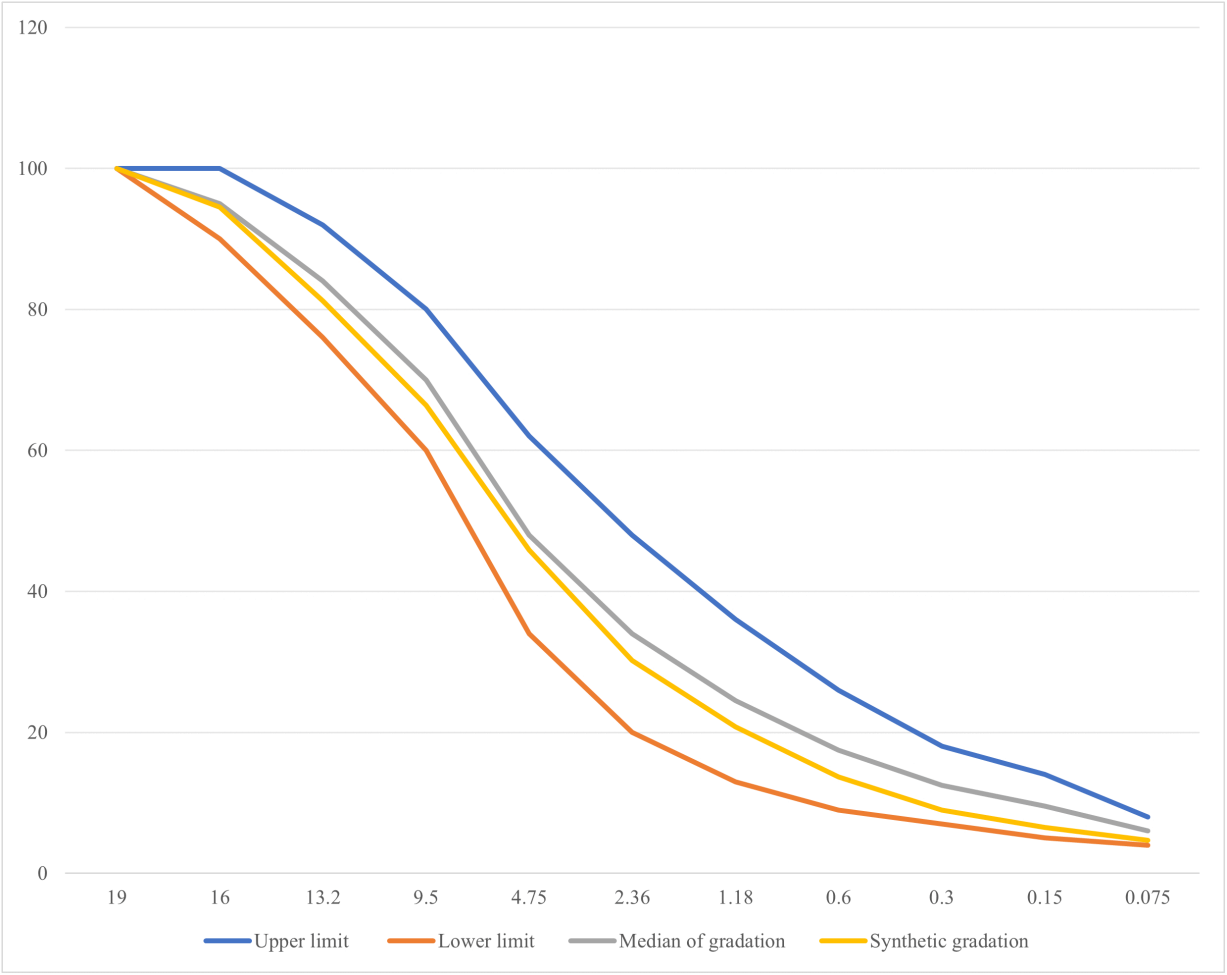
Aggregate gradation curve.

The AC-16 reference aggregate gradation curve is shown in Fig 1.

#### 2.2.2 Rejuvenator content and new-to-old asphalt ratio.

The rejuvenator content was gradually increased from 10% to 12% to 14%, and the technical indexes of the rejuvenated asphalt were tested. It was found that when the content reached 14%, the penetration index met the specification requirements. Meanwhile, according to the most unfavorable factor, the new-to-old asphalt ratio was determined to be 50%:50%, which belongs to the typical design range for high-content RAP recycling studies [14]. The basic performance indexes of the blended new and old asphalt are shown in Table 5.

**Table 5:** Basic performance indexes of blended new and old asphalt.

Test Index	Test Result	Performance Index
Penetration (25°C, 0.1 mm)	92.3	80-100
Softening point (°C)	59.4	>55
Ductility at 15°C (cm)	48.4	>40

#### 2.2.3 Mixture composition.

Based on the basalt reference gradation, the response surface method (Box-Behnken Design, BBD) was used to determine the optimal mixing ratio. The factors were: Factor A: volume ratio of 5–10 mm RAP aggregates (levels: 0%, 50%, 75%); Factor B: volume ratio of 10–20 mm RAP aggregates (levels: 0%, 50%, 75%); Factor C: asphalt-aggregate ratio (levels: 4.0%, 4.5%, 5.0%). With stability, flow value, bulk density, VMA, air void content, and VFA as response values, a regression model was established through 15 groups of Marshall tests. The optimal mix ratio was obtained: RAP aggregates replacement rate of 49.5% and steel slag replacement rate of 50.5% in both 5–10 mm and 10–20 mm fractions, with an asphalt-aggregate ratio of 4.66%. The synthetic gradation of the mixture under this ratio meets the AC-16 gradation requirements, and the performance indexes are shown in Table 6.

**Table 6:** Pavement performance validation results of recycled asphalt mixture.

Test Index	Test Result	Performance Index
Dynamic stability (times/mm)	5112.6	≥2400
Retained stability (%)	89.1	≥80
Retained strength ratio (%)	91.3	≥75
Failure strain (µε)	3325.4	≥2800

### 2.3 Sample preparation

#### 2.3.1 Asphalt sample preparation.

**Fig 2:**
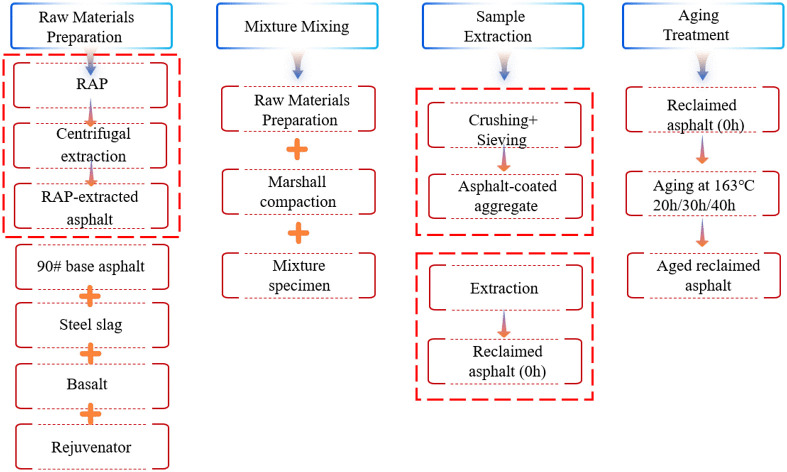
Test samples and preparation process (h = hours).

90# base asphalt is unaged new asphalt. RAP-extracted asphalt is old asphalt directly extracted from raw RAP aggregates, which is un-rejuvenated aged asphalt. The 0h reclaimed asphalt was obtained by extracting the binder from the optimal mixture specimen (already mixed with 14% rejuvenator at 180°C). This sample has experienced process-induced aging during mixing and compaction. This asphalt is the initial reclaimed asphalt after fusion of RAP old asphalt and rejuvenator. The 20h, 30h, and 40h reclaimed asphalt samples were prepared by aging the 0h sample in a thin-film oven at 163°C for 20h, 30h, and 40h respectively, simulating additional aging during service life. The preparation process is shown in Fig 2.

#### 2.3.2 Aggregate sample preparation.

Uncoated aggregates are the original basalt, steel slag, and RAP aggregates before mixture preparation, used to characterize the chemical and mineral properties of the aggregates themselves. Asphalt-coated aggregates are taken from the mixture specimen, and after crushing and sieving, aggregates with particle sizes of 5–10 mm and 10–20 mm are selected to characterize the actual interfacial bonding state between asphalt and aggregates. The same particle size range is selected to ensure test operability and comparability.

### 2.4 Test methods

#### 2.4.1 Asphalt characterization analysis.

Fourier transform infrared spectroscopy (FTIR) is an important means of identifying asphalt chemical structure and functional group changes by analyzing molecular vibration absorption characteristics. A HF-03 asphalt rapid analyzer was used to perform FTIR tests on 90# base asphalt, RAP-extracted asphalt, reclaimed asphalt, and reclaimed asphalt with different aging times. The instrument parameters were set as follows: resolution 4 cm ⁻ ¹, scanning range 500–4000 cm ⁻ ¹, preheating temperature 30°C, and the number of scans was 32 times.

#### 2.4.2 Aggregate characterization methods.

No permits were required. All experiments were conducted in the laboratory.

**2.4.2.1 XRF test:** An X-ray fluorescence spectrometer was used to analyze the chemical elements and oxide compositions of the three aggregates. Focus was placed on seven rock oxides: SiO₂, Al₂O₃, Fe₂O₃, CaO, MgO, Na₂O, K₂O, as well as characteristic components such as TiO₂, MnO, P₂O₅, SO₃, and Cl.

**2.4.2.2 XRD test:** An X-ray diffractometer was used to analyze the mineral phase composition and microstructural parameters of the three aggregates before and after asphalt coating. MDI Jade software was used for phase retrieval and matching to calculate crystallite size (D), micro-strain (ε), dislocation density (ρ), and interplanar spacing (d). The calculation formulas are as follows:


$$\begin{array}{c} \text{Crystallite size}:\;D=\frac{K\lambda }{\beta \text{cos}\theta } \end{array}$$ (1)

$$\begin{array}{c} \text{Micro}-\text{strain}:\;\beta \text{cos}\theta =\frac{K\lambda }{D}+\text{4}\varepsilon \text{sin}\theta \end{array}$$ (2)

$$\begin{array}{c} \text{Dislocation density}:\;\rho =\frac{\text{1}\text{4}.\text{4}}{\sqrt{\text{3}}D^{\text{2}}} \end{array}$$ (3)

$$\begin{array}{c} \text{Interplanar spacing}:\;d=\frac{\lambda }{\text{2}\text{sin}\theta } \end{array}$$ (4)

Where: λ is the X-ray wavelength; K is the shape factor (usually 0.9); β is the full width at half maximum of the diffraction peak (radians); θ is the Bragg angle (radians).

**2.4.2.3 SEM-EDS test:** A scanning electron microscope was used to observe the surface morphology of the three aggregates before and after asphalt coating and the interfacial bonding state between asphalt and aggregates, with magnification ranging from 200 to 3000 times. An energy dispersive spectrometer was used to analyze the distribution of elements in the surface micro-areas.

#### 2.4.3 Characterization methods for interfacial adhesion properties.

**2.4.3.1 Contact angle test:** An XG-CAM contact angle measuring instrument was used to test the contact angles of different asphalts (90#, RAP-extracted asphalt, 0-40h reclaimed asphalt) and different aggregates (RAP, basalt, steel slag) using distilled water, formamide, and diiodomethane as probe liquids. The surface energy parameters of the three test liquids are shown in Table 7.

**Table 7:** Surface free energy parameters of three test liquids (unit: mJ/m²).

Test Liquid	Surface Energy $\gamma_{l}$	Dispersive Component $\overset{d}{\underset{l}{\gamma }}$	Polar Component $\overset{p}{\underset{l}{\gamma }}$
Distilled water	72.80	21.80	51.00
Formamide	58.00	39.00	19.00
Diiodomethane	50.80	48.50	0.00

Based on the Young-Dupré equation and the Owens-Wendt method, the surface energy parameters and adhesion work between asphalt and aggregates were calculated [5,6]. The calculation process is as follows:

The solid surface free energy is composed of the dispersive component $\overset{d}{\underset{s}{\gamma }}$ and the polar component $\overset{p}{\underset{s}{\gamma }}$,satisfying:


$$\begin{array}{c} \gamma_{s}=\overset{d}{\underset{s}{\gamma }}+\overset{p}{\underset{s}{\gamma }} \end{array}$$ (5)

By measuring the contact angle θ of the test liquid on the solid surface, the Young equation and the Owens-Wendt model are combined:


$$\begin{array}{c} \gamma_{l}(1+cos\theta )=2\left(\sqrt{\overset{d}{\underset{s}{\gamma }}\overset{d}{\underset{l}{\gamma }}}+\sqrt{\overset{p}{\underset{s}{\gamma }}\overset{p}{\underset{l}{\gamma }}}\right) \end{array}$$ (6)

Where $\gamma_{l}$, $\overset{d}{\underset{l}{\gamma }}$, $\overset{p}{\underset{l}{\gamma }}$ are the known surface energy parameters of the test liquid (Table 7). Through the contact angle data of the three test liquids, $\overset{d}{\underset{s}{\gamma }}$ and $\overset{p}{\underset{s}{\gamma }}$ can be solved.

The adhesion work $W_{as}$ between asphalt and aggregate is calculated by the following formula:


$$\begin{array}{c} W_{as}=2\left(\sqrt{\overset{d}{\underset{a}{\gamma }}\overset{d}{\underset{s}{\gamma }}}+\sqrt{\overset{p}{\underset{a}{\gamma }}\overset{p}{\underset{s}{\gamma }}}\right) \end{array}$$ (7)

Where $\gamma_{a}$, $\gamma_{s}$ are the surface free energies of asphalt and aggregate, respectively.

**2.4.3.2 AFM test:** A MultiMode 8 atomic force microscope was used to scan the micromorphology of different asphalts (90#, RAP, 0-40h reclaimed asphalt) in tapping mode, with a scanning range of 20μm × 20μm. The root mean square roughness (Rq) and adhesion force data were obtained. The calculation formula for root mean square roughness is:


$$\begin{array}{c} R_{q}=\sqrt{\frac{\int \int [h(x,y)-h_{\text{0}}{]}^{\text{2}}d_{s}}{\int \int d_{s}}} \end{array}$$ (8)

Where: s is the AFM image scanning area; h(x,y) is the height function of the morphology (nm); $h_{\text{0}}$ is the reference height (nm).

**2.4.3.3 Pull-off test:** A B225 multifunctional direct tensile tester was used to test the tensile strength (TS) of six asphalt-aggregate combinations (BB: basalt-basalt, RR: RAP-RAP, SS: steel slag-steel slag, BR: basalt-RAP, BS: basalt-steel slag, RS: RAP-steel slag) at 25°C. The asphalt film thickness was controlled at 25 ± 5 μm, and the loading rate was 1 mm/min.


$$\begin{array}{c} TS=\frac{F}{S} \end{array}$$ (9)

Where: *TS* is the tensile strength of the asphalt-aggregate interface (MPa); *F* is the peak pull-off force at the asphalt-aggregate interface (N); *S* is the area of the asphalt-aggregate interface (mm²).

## 3. Results and analysis

### 3.1 FTIR-based analysis of asphalt chemical characteristics

#### 3.1.1 Infrared spectroscopy analysis.

**Fig 3:**
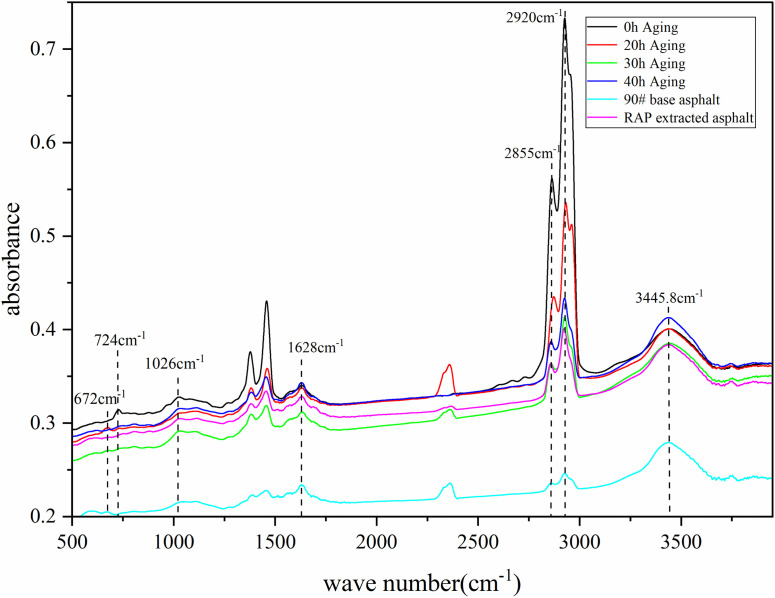
FTIR spectra comparison of different asphalts.

Fig 3 shows the FTIR spectra comparison of six asphalts. The detailed FTIR spectra data are provided in S1 File. Overall, the FTIR spectra of the six asphalts show small differences, with the main characteristic peak positions basically consistent and no new functional groups generated, indicating that aging and rejuvenation processes have little effect on the functional group backbone structure of asphalt.

The absorption peak near 1628 cm ⁻ ¹ corresponds to the stretching vibration of the carbon-carbon double bond in the benzene ring, indicating the presence of aromatic structures. The intensity of the sulfoxide vibration peak near 1026 cm ⁻ ¹ increases due to oxidative condensation and dehydrogenation reactions during aging [8]. Strong absorption peaks appear near 2920 cm ⁻ ¹ and 2855 cm ⁻ ¹, corresponding to the stretching vibrations of C-H bonds in methylene and aliphatic methyl groups. The peak at 3445.8 cm ⁻ ¹ corresponds to O-H stretching vibration, confirming that all asphalts contain hydroxyl components.

Notably, the 0h reclaimed asphalt shows an absorption peak at 724 cm ⁻ ¹, and the 20h reclaimed asphalt shows an absorption peak at 672 cm ⁻ ¹. The 724 cm ⁻ ¹ peak corresponds to the out-of-plane rocking vibration of long-chain methylene groups. As the aging time increases, the intensity of this peak gradually weakens, indicating that aging conditions reduce the content of long-chain methylene groups. The 672 cm ⁻ ¹ absorption peak is located in the fingerprint region and may be related to the out-of-plane bending vibration of olefins or aromatics, suggesting the formation of new unsaturated structures during aging.

#### 3.1.2 Quantitative analysis of functional group indices.

(1) Aromatic Index (AI): Characterizes the relative content of aromatic structures in asphalt, calculated as:

$$\begin{array}{c} AI=\frac{A_{=CH}}{A_{C-H}} \end{array}$$ (10)

Where: $A_{=CH}$ is the area of the aromatic molecular vibration peak (around 1600 cm ⁻ ¹); $A_{C-H}$ represents the sum of the areas of C-H bond vibration peaks, including 2920 cm ⁻ ¹ (asymmetric stretching of methylene), 2855 cm ⁻ ¹ (symmetric stretching of methylene), 1450 cm ⁻ ¹ (bending of methyl and methylene), 1375 cm ⁻ ¹ (bending of methyl), etc.

(2) Sulfoxide Index (SI): Characterizes the degree of asphalt oxidation, reflecting changes in sulfur-containing functional groups, calculated as:

$$\begin{array}{c} SI=\frac{A_{S=O}}{A_{C-H}} \end{array}$$ (11)

Where: $A_{S=O}$ is the area of the sulfoxide vibration peak (around 1030 cm ⁻ ¹).

(3) Asymmetry Index (AsI): Characterizes the degree of branching and disorder of aliphatic structures, calculated as:

$$\begin{array}{c} AsI=\frac{A_{2920}+A_{2855}}{A_{C-H}} \end{array}$$ (12)

Where: $A_{2920}$ is the area of the asymmetric stretching vibration peak of methylene C-H at 2920 cm ⁻ ¹; $A_{2855}$ is the area of the symmetric stretching vibration peak of methylene C-H at 2855 cm ⁻ ¹.

**Fig 4:**
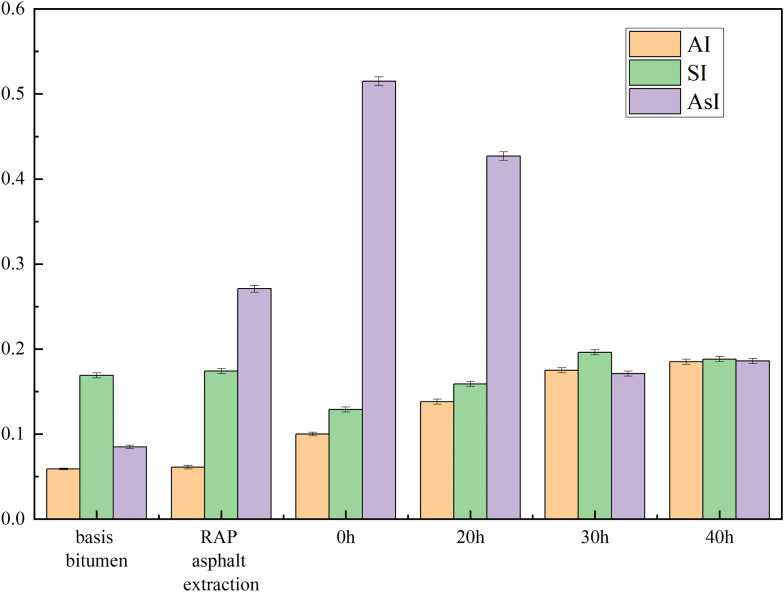
Comparison of functional group indices of different asphalts.

The peak areas of each characteristic peak were calculated using OMNIC software, with automatic baseline correction and manual adjustment of integration ranges based on actual peak shapes to ensure accuracy. The calculated functional group indices of the six asphalts are shown in Fig 4.

#### 3.1.3 Analysis of functional group evolution.

From Fig 4, it can be seen that the aromatic index (AI) of 90# base asphalt and RAP-extracted asphalt is relatively close (0.059 and 0.061), indicating that aging has limited effect on the aromatic skeleton. The sulfoxide index (SI) of RAP-extracted asphalt is slightly higher than that of base asphalt (0.174 vs. 0.169), reflecting an increased degree of oxidation [8]. The asymmetry index (AsI) of RAP-extracted asphalt is significantly higher than that of base asphalt (0.271 vs. 0.085), indicating the fracture of long-chain alkanes and an increase in the relative content of short-chain alkanes during aging [9,16].

The functional group indices of reclaimed asphalt show regular evolution with aging time. AI increases from 0.100 at 0h to 0.185 at 40h, an increase of 85%, reflecting the relative enrichment of aromatic structures, increased asphalt viscosity, and decreased flexibility. SI first increases and then decreases, rising by 52% from 0h to 30h and decreasing by 4% from 30h to 40h. In the first 30h, oxidation reactions dominate, and asphalt polarity increases; after 30h, the rejuvenator dilutes the sulfoxide concentration, and oxidation and rejuvenation reach a dynamic equilibrium. AsI first decreases sharply and then increases slightly, decreasing by 67% from 0h to 30h and increasing by 9% from 30h to 40h. In the first 30h, long-chain fracture leads to increased asphalt brittleness; after 30h, the rejuvenator replenishes some long-chain structures, and flexibility is slightly restored^16^.

30h is the critical point for functional group evolution. After 30h, the changes in each index slow down, indicating that the diffusion of the rejuvenator and the aging reaction tend to reach equilibrium.

### 3.2 Chemical and mineralogical characteristics of aggregates

#### 3.2.1 XRF-based chemical composition analysis.

**Fig 5:**
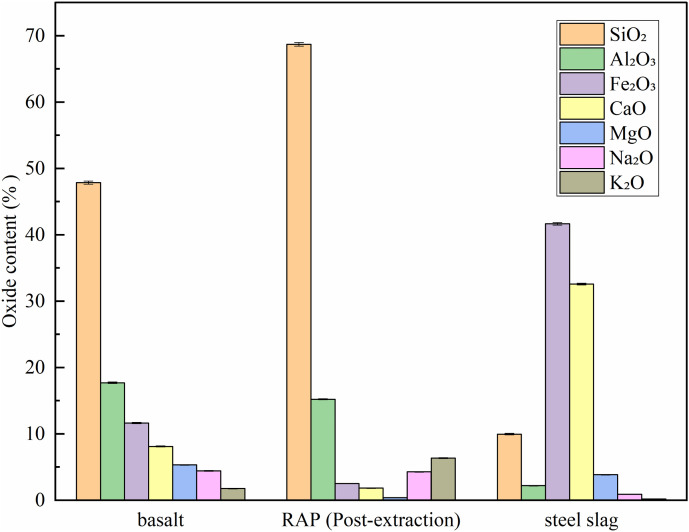
Main oxide contents of three aggregates.

Fig 5 shows the main oxide contents of the three aggregates. Basalt has a balanced chemical composition, with SiO₂, Al₂O₃, Fe₂O₃, and CaO contents of 47.88%, 17.71%, 11.64%, and 8.12%, respectively, making it a typical basic rock. RAP aggregates have a high SiO₂ content of 68.71% and a K₂O + Na₂O content of 10.65%, with very low CaO, MgO, and Fe₂O₃ contents, making it an acidic granite. Steel slag exhibits high calcium and high iron characteristics, with CaO content of 32.58%, Fe₂O₃ content of 41.66%, and SiO₂ content of only 9.97%, departing from the natural rock category.

**Fig 6:**
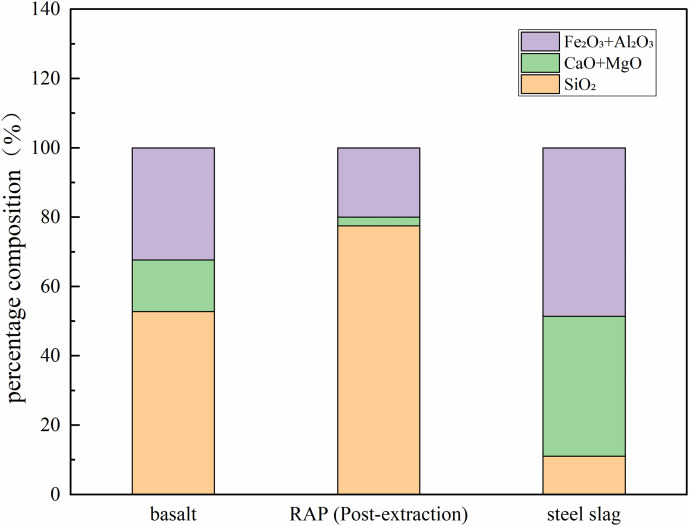
Stacked diagram of acidic and alkaline components of three aggregates.

Fig 6 shows the stacked diagram of acidic and alkaline components of the three aggregates. To determine the lithological attribution and reactivity of the aggregates, the main oxides were grouped into three comprehensive indicators: SiO₂ (acidic component), CaO + MgO (alkaline component), and Fe₂O₃ + Al₂O₃ (ferric-aluminous component), and normalized to 100%.

The three aggregates show significant differences in chemical composition. Basalt has SiO₂ accounting for about 52.8%, with CaO + MgO and Fe₂O₃ + Al₂O₃ totaling about 47%, belonging to basic rock, with a surface rich in metal cation sites, favorable for physical adsorption and polar interactions. RAP aggregates have a high SiO₂ content of 77.5%, belonging to acidic granite, with weak direct affinity to asphalt, but the residual aged film on its surface will dominate the actual interfacial behavior. Steel slag exhibits high calcium and high iron characteristics, with Fe₂O₃ + Al₂O₃ accounting for 48.6%, CaO + MgO accounting for 40.4%, and SiO₂ accounting for only 11.0%, belonging to typical artificial metallurgical slag, with abundant alkaline active sites on the surface, prone to chemical bonding with asphalt.

In summary, basalt is dominated by physical adsorption, RAP is dominated by residual film activation, and steel slag is dominated by chemical bonding.

#### 3.2.2 XRD-based mineral phase analysis.

The main mineral phases of the three aggregates and their changes before and after asphalt coating are shown in Table 8.

**Table 8:** Changes in main mineral phases of different aggregates before and after asphalt coating.

Aggregate Type	Mineral Phase	Before Coating (%)	After Coating (%)	Change	Possible Mechanism
Basalt	Anorthite	68.4	60.5	↓7.9	Covered with asphalt, the signal is weakened.
	Clinopyroxene	21.8	28.7	↑6.9	Polar adsorption, surface enrichment
RAP	Quartz	58.1	53.3	↓4.8	Covered with asphalt, the signal is weakened.
	Albite	25.1	30.0	↑4.9	Selective adsorption, surface enrichment
	Mica	8.9	16.0	↑7.1	Layered structure exposure, surface enrichment
Steel slag	RO phase	52.3	39.4	↓12.9	Consumption by saponification reaction
	Brownmillerite	34.0	40.6	↑6.6	Reaction generation
	Dicalcium silicate	13.7	16.9	↑3.2	Recrystallization/enrichment
	Tricalcium silicate	–	3.0	↑3.0	New phase generation

Asphalt preferentially adsorbs on the surface of clinopyroxene in basalt. In RAP, the quartz signal weakens, while albite and mica become enriched on the surface. In steel slag, the RO phase decreases, while brownmillerite and silicate new phases are generated, confirming the chemical bonding between asphalt and steel slag [13].

#### 3.2.3 XRD-based microstructural evolution analysis.

**Fig 7:**
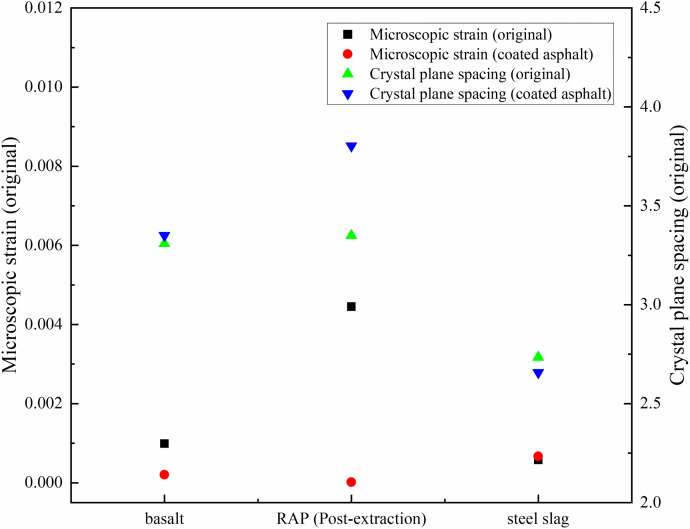
Changes in micro-strain and interplanar spacing of three aggregates before and after asphalt coating (percentage changes are relative to uncoated aggregate baseline).

The percentage changes shown in Fig 7 are calculated relative to the uncoated aggregate as baseline. For example, the micro-strain of uncoated basalt is 9.92 × 10 ⁻ ⁴; after asphalt coating, it decreases to 2.03 × 10 ⁻ ⁴, representing a decrease of 79.5%. Similar absolute baseline values have been added for RAP aggregates and steel slag.

Fig 7 shows the changes in micro-strain and interplanar spacing of the three aggregates before and after asphalt coating, revealing three distinctly different evolution patterns.

After basalt coating, the micro-strain sharply decreases by 79.5%, and the interplanar spacing slightly increases by 1.25%. This indicates that asphalt mainly acts as a physical filler, releasing the internal stress of the surface mineral layer, which is a typical manifestation of physical adsorption dominance.

After RAP aggregate coating, the micro-strain almost returns to zero (a decrease of 99.6%), and the interplanar spacing sharply increases by 13.5%. This is direct microscopic evidence of the rejuvenator penetrating the aged asphalt network, achieving the “swelling” and bonding capacity recovery of the old asphalt.

After steel slag coating, the micro-strain increases by 15.5%, and the interplanar spacing decreases by 2.82%. This is a typical characteristic of chemical bonding, where the saponification reaction disrupts the integrity of the original mineral lattice while generating a denser new interfacial phase [12].

#### 3.2.4 SEM-EDS morphological observation.

**Fig 8:**
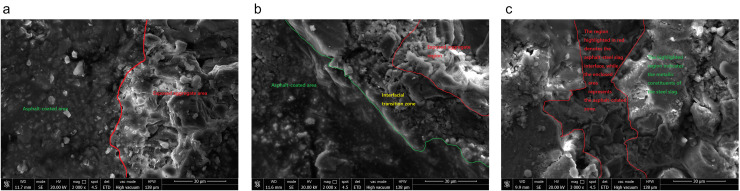
Surface morphology of three aggregates and interfacial state after asphalt coating.

**Basalt RAP Steel slag:** Fig 8 shows the interfacial regions between different aggregates and asphalt. The observations are as follows:

A clear interface line exists between asphalt and basalt, with no gaps or peeling. Asphalt extends into the mineral micro-cracks in a “tongue-like” manner, and no reaction product layer is observed at the interface. This is a typical morphology dominated by physical adsorption [8].

A transition layer with a thickness of several hundred nanometers to micrometers exists between steel slag and asphalt. The surface layer of steel slag shows an “etched” or “eroded” morphology, with fine reaction pits and newly generated crystals visible. This is direct evidence of chemical bonding and interface reconstruction [13].

The RAP aggregates interface is clearly divided into three layers: the mineral substrate, the intermediate residual layer, and the new asphalt layer. Parts of the intermediate residual layer are well fused with the new asphalt, with a blurred interface; other parts still retain the characteristic cracking texture of the aged film, indicating that the rejuvenator has begun to act but the activation is not yet complete. This is a typical morphology of residual film activation and double-layer fusion [9].

### 3.3 Interfacial adhesion mechanism

#### 3.3.1 Surface energy-based analysis of asphalt-aggregate adhesion properties.

**Fig 9:**
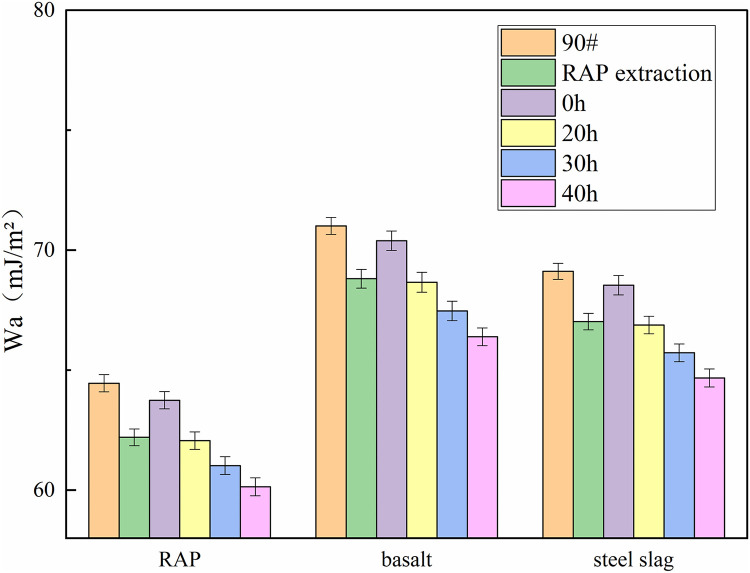
Comparison of adhesion work between different asphalts and aggregates.

The surface energy parameters of six asphalts and three aggregates were measured through contact angle tests, and the calculated adhesion work between asphalts and aggregates is shown in Fig 9.

Regarding the effect of aggregate type on adhesion work, regardless of the asphalt type, the adhesion work follows the pattern of basalt > steel slag > RAP aggregates, consistent with existing research results [11]. Taking 90# base asphalt as an example, the adhesion work of basalt is 71.01 mJ/m^2^, steel slag is 69.12 mJ/m^2^, and RAP is 64.46 mJ/m^2^.

Regarding the effect of asphalt type on adhesion work, taking basalt as an example, the adhesion work of 90# base asphalt is 71.01 mJ/m^2^, while that of RAP-extracted asphalt is 68.81 mJ/m^2^ (a decrease of 3.0%). The adhesion work of 0h reclaimed asphalt is 70.40 mJ/m^2^, close to the level of base asphalt. The adhesion work of 40h reclaimed asphalt decreases to 66.40 mJ/m^2^ (a cumulative decrease of 5.7%).

The core of this study is the RAP-steel slag dual solid waste recycling system. Taking reclaimed asphalt (0h) as an example, its adhesion work with steel slag is 68.54 mJ/m^2^, and with RAP aggregate is 63.75 mJ/m^2^, a difference of about 4.79 mJ/m^2^. This difference originates from the different surface characteristics of the two aggregates: the steel slag surface is rich in alkaline active sites (CaO, Fe₂O₃), which can undergo chemical bonding with reclaimed asphalt; while the RAP aggregate surface has a residual aged asphalt film. Although activated by the rejuvenator, its cohesive strength is still lower than the chemical bonding interface of steel slag-asphalt. Therefore, in the dual solid waste recycling system, steel slag primarily undertakes the enhancement role of chemical bonding and mechanical interlocking, while RAP aggregate achieves the re-bonding of old asphalt through residual film activation. The two synergistically constitute a multiple interface enhancement mechanism of “physical adsorption, chemical bonding, and mechanical interlocking.”

#### 3.3.2 AFM-based analysis of asphalt micromorphology and adhesion force.

**Fig 10:**
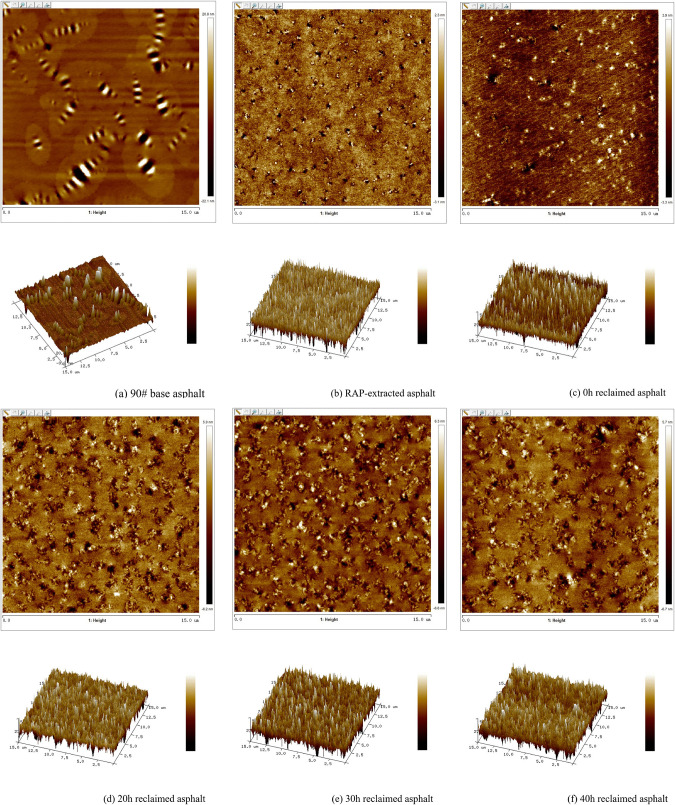
AFM morphology and mechanical properties of different asphalts.

Fig 10 shows the AFM morphology images (2D and 3D) of six asphalts, clearly showing the “bee-like structure” on the asphalt microscopic surface and its evolution pattern.

90# base asphalt exhibits a clear three-phase structure with a uniformly distributed “bee-like structure,” individual and distinct. RAP-extracted asphalt shows a completely different micromorphology from base asphalt. The “bee-like structure” becomes fragmented and dispersed, with no obvious independent long individual structures. The number of needle-like “sharp and fine” structures increases, and the surface undulation decreases. Combined with the FTIR analysis showing the disappearance of the characteristic peak of long-chain alkyl compounds at 722 cm ⁻ ¹, this indicates that the high temperature and solvent during the extraction process destroyed the wax crystal structure, leading to the fragmentation of the “bee-like structure.” The periphase basically disappears, forming a relatively uniform “two-phase structure.” According to the evolution pattern of the “bee-like structure,” the aging process of reclaimed asphalt can be divided into three stages: initial stage (0-20h): the wax crystals in the original “bee-like structure” gradually twist and decompose, some light components begin to transform, the boundaries become blurred, slight fusion occurs, and the periphase gradually disappears. Middle stage (20-30h): the concentration of asphaltene aggregation reaches a critical point, reorganizing into a state where black aggregates adhere to white aggregates, and asphaltene begins to form a continuous network structure. Late stage (30-40h): asphaltene becomes the dominant phase, forming a highly continuous, high-modulus network structure. The soft phase is almost “engulfed,” microscopic phase separation disappears, and overall hardening occurs.

**Fig 11:**
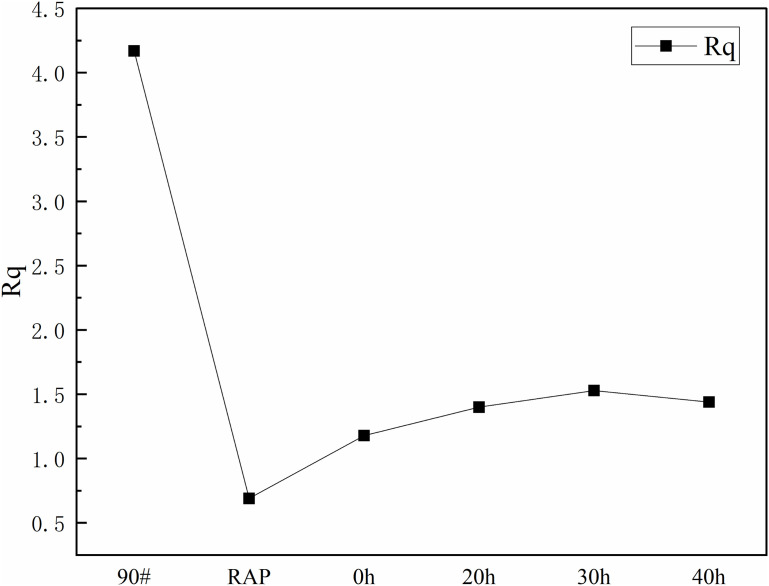
Root mean square roughness of different asphalts.

Fig 11 shows the root mean square roughness (Rq) test results of different asphalts. The Rq of 90# base asphalt (4.17 nm) is much higher than that of RAP-extracted asphalt (0.69 nm). This is because during long-term aging, the volatilization of light components and the aggregation of asphaltenes lead to surface hardening and densification, significantly reducing the roughness. The Rq of reclaimed asphalt first increases and then decreases with aging time: 1.18 nm at 0h, increasing to 1.40 nm at 20h, reaching a peak of 1.53 nm at 30h, and then decreasing to 1.44 nm at 40h. This trend is highly consistent with the evolution pattern of the AsI index, indicating an inherent correlation between aliphatic structure and micromorphological roughness [16].

**Fig 12:**
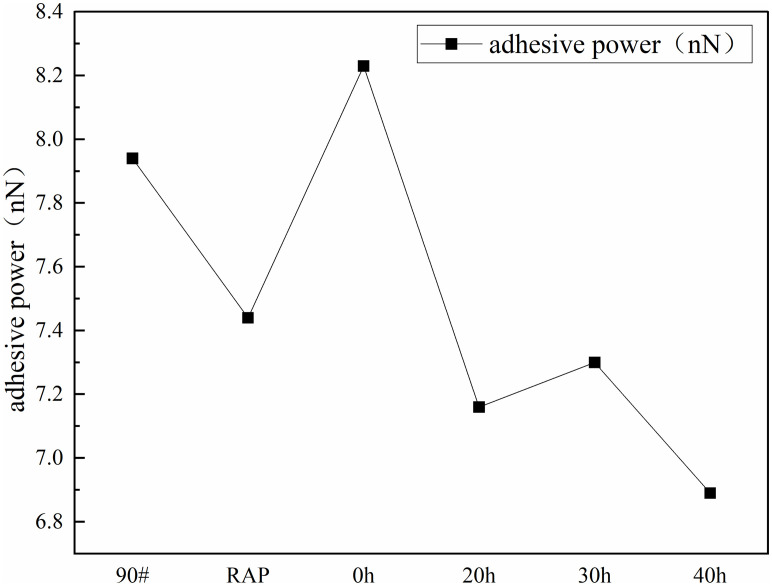
Adhesion force of different asphalts.

Fig 12 shows the adhesion force test results. The adhesion force of 90# base asphalt is 7.94 nN, and that of RAP-extracted asphalt is 7.44 nN, a decrease of 6.3%. The adhesion force of 0h reclaimed asphalt is the highest (8.23 nN), even exceeding that of base asphalt, indicating that the rejuvenator effectively restored the bonding performance of aged asphalt. Subsequently, the adhesion force shows a trend of “rapid decrease - brief stabilization - continuous deterioration”: decreasing to 7.16 nN at 20h, slightly increasing to 7.30 nN at 30h, and then decreasing to 6.89 nN at 40h. This may be related to the diffusion of the rejuvenator in the aged asphalt and the evolution of polar functional groups during the re-aging process [15].

The adhesion force is not simply positively correlated with roughness. The 0h reclaimed asphalt has low roughness but the highest adhesion force, indicating that the chemical composition of asphalt has a greater influence on the adhesion force than surface morphology. The change trend of adhesion force is consistent with the evolution of AI and SI indices, further confirming the decisive role of chemical structure on nanoscale bonding performance [17].

#### 3.3.3 Pull-off test-based analysis of interfacial tensile strength.

**Fig 13:**
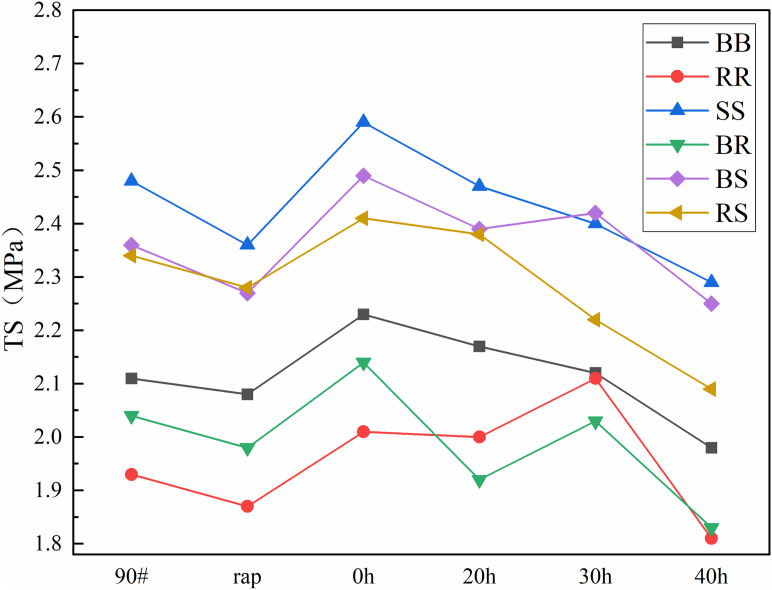
Comparison of pull-off strength TS.

Fig 13 shows the pull-off strength test results of different asphalt-aggregate combinations at 25°C. Three aggregates (basalt, steel slag, RAP aggregates) and six asphalts (90# base, RAP-extracted, 0h/20h/30h/40h aged reclaimed asphalt) were selected to form six interface combinations: BB (basalt-basalt), RR (RAP-RAP), SS (steel slag-steel slag), BR (basalt-RAP), BS (basalt-steel slag), RS (RAP-steel slag).

(1) Effect of asphalt type on pull-off strength. For the same aggregate combination, the pull-off strength of 90# base asphalt is higher than that of RAP-extracted asphalt. Taking the SS combination as an example, the TS of 90# base asphalt is 2.48 MPa, while that of RAP-extracted asphalt is 2.36 MPa, a decrease of 4.8%. This is consistent with the trend of adhesion work in Section 3.3.1, indicating that aging leads to a decrease in asphalt bonding performance.

The pull-off strength of 0h reclaimed asphalt is higher than that at other aging times, even exceeding that of base asphalt (BB: 2.23 vs. 2.11; SS: 2.59 vs. 2.48). This is because the aromatic and saturated components in the rejuvenator supplement the light components lost from the aged asphalt, dilute the aggregated asphaltenes, and restore the fluidity of asphalt molecular chains and the activity of polar functional groups, thereby enhancing the physical adsorption and chemical bonding ability between asphalt and aggregates. However, as aging time increases, the light components in the rejuvenator volatilize again, and asphaltenes aggregate again, leading to a continuous decrease in pull-off strength. By 40h, it has fallen below the level of RAP-extracted asphalt.

(2) Effect of aggregate type on pull-off strength. The SS combination has the highest strength, reaching 2.48 MPa with 90# base asphalt and 2.59 MPa with 0h reclaimed asphalt. This benefits from the mechanical interlocking provided by the rough and porous surface morphology of steel slag, as well as the chemical bonding between steel slag and asphalt [12]. The BS combination ranks second, with 2.36 MPa under 90# base asphalt, indicating that the combination of steel slag and basalt also has excellent interfacial performance. The BB combination is in the middle, with 2.11 MPa under 90# base asphalt, showing stable and reliable interfacial performance of basalt. The RR combination has the lowest strength, only 1.93 MPa under 90# base asphalt. This is because the residual aged asphalt film on the RAP aggregate surface has low cohesive strength, and interface failure may occur within the residual film rather than at the asphalt-aggregate interface [9]. The pull-off strengths of the BR and RS combinations are between those of their respective single combinations, indicating that the performance of heterogeneous interfaces can be balanced by optimizing the mixing ratio.
(3) Effect of aging time on pull-off strength. In the BB, SS, and BS combinations, the pull-off strength shows a monotonically decreasing trend from 0h to 40h, consistent with the change pattern of adhesion work in Section 3.3.1. This is related to the volatilization of light components and the decrease in polar functional groups during asphalt aging. In the RR combination, the pull-off strength at 30h (2.11 MPa) is higher than that at 20h (2.00 MPa), showing a slight recovery. Combined with the FTIR analysis in Section 3.1.2, at 30h, SI reaches its peak and AsI reaches its valley, indicating a transition in the chemical structure of asphalt. At the same time, AFM shows that at 30h, the asphalt microstructure forms a continuous network, which may enhance mechanical interlocking. Similar incomplete monotonic phenomena are also observed in the RS and BS combinations, indicating that the response of steel slag-containing interfacial structures to the degree of asphalt aging is more complex, confirming the multi-scale fusion mechanism of physical adsorption, chemical bonding, and mechanical interlocking of steel slag.

#### 3.3.4 Correlation analysis of adhesion parameters and macroscopic performance.

Combining adhesion work, AFM adhesion force, and pull-off strength, it can be found that there are good correlations among the three, collectively forming a complete evidence chain from thermodynamics, nanomechanics, to macro-mechanics.

**Fig 14:**
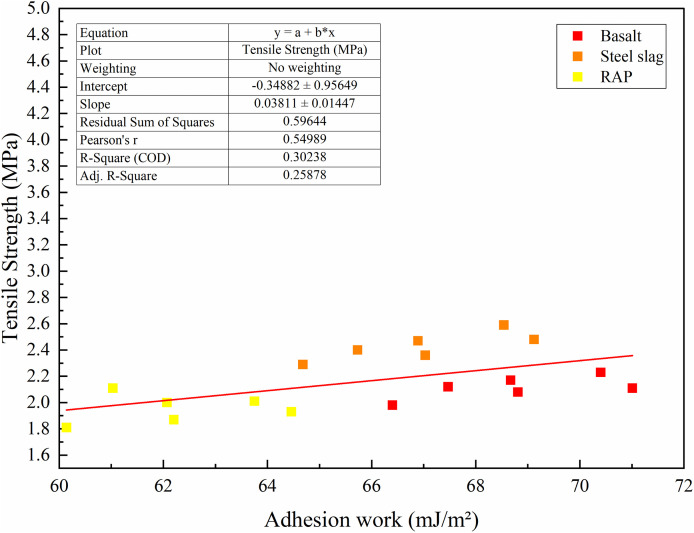
Correlation between adhesion work and pull-off strength (N = 18, R^2^ = 0.92, p < 0.01).

(1) **Correlation between adhesion work and pull-off strength.** Fig 14 is a scatter plot of the correlation between adhesion work and pull-off strength, where each point represents an asphalt-aggregate combination (6 asphalts × 3 aggregates = 18 data points).

Adhesion work and pull-off strength show a significant positive correlation, with a linear fitting R^2^ of 0.92, indicating that surface energy theory can effectively predict the macroscopic interfacial performance of asphalt and aggregates [17]. It is worth noting that the steel slag combinations are mostly located above the fitting line, with their pull-off strength higher than the predicted value of adhesion work. Taking 90# base asphalt as an example, the adhesion work of steel slag (69.12 mJ/m^2^) is slightly lower than that of basalt (71.01 mJ/m^2^), but its pull-off strength (2.48 MPa) is higher than that of basalt (2.11 MPa). This “anomalous” phenomenon indicates that adhesion work mainly reflects reversible physical adsorption contributions, while pull-off strength also includes the irreversible chemical bonding (saponification reaction) on the steel slag surface and the mechanical interlocking contribution provided by its rough and porous surface. The adhesion work and pull-off strength of RAP are both the lowest, indicating that although the residual aged film increases the polar component, its cohesive strength is insufficient, making it a weak link in the interface.

**Fig 15:**
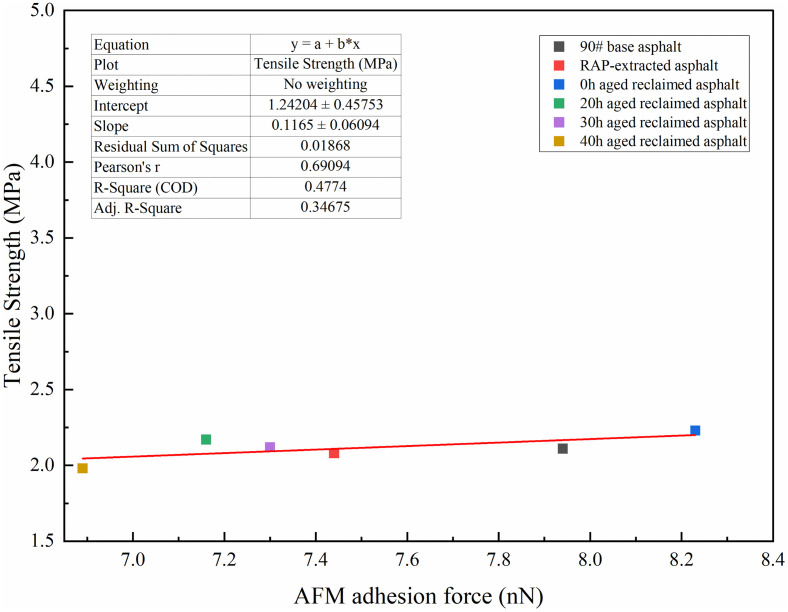
Correlation between AFM adhesion force and pull-off strength (N = 6, R^2^ = 0.89, p < 0.01).

(2) **Correlation between AFM adhesion force and pull-off strength.** Fig 15 is a scatter plot of the correlation between AFM adhesion force and pull-off strength, where each point represents an asphalt (6 asphalts), with the aggregate fixed as basalt.

AFM adhesion force and pull-off strength show a good positive correlation, with a linear fitting R^2^ of 0.89. This indicates that the stronger the nanoscale bonding performance of the asphalt itself, the higher the macroscopic interfacial strength formed with the aggregate [15]. The 0h reclaimed asphalt has the highest AFM adhesion force (8.23 nN) and pull-off strength (2.23 MPa), both located above the fitting line, confirming that the rejuvenator effectively restored the bonding performance of aged asphalt. As aging time increases, the AFM adhesion force and pull-off strength synchronously decrease, reaching 6.89 nN and 1.98 MPa at 40h, respectively, indicating that continuous aging causes irreversible damage to asphalt bonding performance. The change trend of AFM adhesion force is completely consistent with the adhesion work in Section 3.3.1, both reflecting the performance evolution pattern during asphalt aging and rejuvenation.

(3) **Comprehensive conclusion on adhesion parameters.** The three parameters of adhesion work (thermodynamics), AFM adhesion force (nanomechanics), and pull-off strength (macro-mechanics) show consistent evolution patterns during asphalt aging and rejuvenation, collectively forming an evidence chain from thermodynamics, nanomechanics, to macro-mechanics. Pull-off strength is a comprehensive manifestation of physical adsorption, chemical bonding, and mechanical interlocking, and the contribution of aggregate surface morphology cannot be ignored. The phenomenon of “lower adhesion work but higher pull-off strength” of steel slag reveals the synergistic mechanism of physical adsorption, chemical bonding, and mechanical interlocking, providing a theoretical basis for the selection of high-performance aggregates.

## 4. Conclusions

(1) The interfacial adhesion modes of the three aggregates are considerably different. Compared with steel slag and RAP, basalt is dominated by physical adsorption. Asphalt fills surface defects, causing micro-strain to decrease by 79.5%, forming a dense physical interface. Steel slag is dominated by the synergy of chemical bonding and mechanical interlocking. The saponification reaction generates a new interfacial phase, causing micro-strain to increase by 15.5%, while the rough and porous surface provides mechanical anchoring. RAP aggregates are dominated by residual film activation. The rejuvenator swells the aged film, increasing the interplanar spacing by 13.5%.
(2) The phenomenon of “lower adhesion work but higher pull-off strength” of steel slag reveals the synergistic enhancement mechanism of physical adsorption, chemical bonding, and mechanical interlocking. The adhesion work of steel slag (69.12 mJ/m²) is slightly lower than that of basalt (71.01 mJ/m²), but its pull-off strength (2.48 MPa) is higher than that of basalt (2.11 MPa). This is because adhesion work mainly reflects reversible physical adsorption and chemical adsorption contributions, while pull-off strength also includes the irreversible chemical bonding (saponification reaction) on the steel slag surface and the mechanical interlocking contribution provided by its rough and porous surface.
(3) The evolution pattern of interfacial performance during the re-aging process of reclaimed asphalt is revealed. With increasing aging time, the aromatic index (AI) continuously increases (by 85%), and asphalt viscosity increases. The sulfoxide index (SI) first increases and then decreases, with 30h being the critical point for the balance between oxidation and rejuvenation. The asymmetry index (AsI) first decreases and then increases, reflecting the dynamic balance between long-chain fracture and rejuvenator replenishment. The three parameters of adhesion work, AFM adhesion force, and pull-off strength show consistent evolution patterns during aging and rejuvenation, with performance starting to deteriorate after 30h. A multi-scale interfacial performance evaluation system from thermodynamics to macro-mechanics is established (R² = 0.92 and 0.89, with N = 18 and N = 6 respectively, p < 0.01, respectively).
(4) The residual aged film on RAP aggregates can be activated by the rejuvenator. XRF detects SO₃ and Cl elements, SEM observes a three-layer structure, and XRD shows a 13.5% increase in interplanar spacing. Multi-scale evidence confirms that the rejuvenator can effectively restore the bonding performance of aged asphalt.

## Supporting information

S1 File Raw FTIR data of aged and rejuvenated asphalt. (XLSX)
